# Crystal structure of μ-oxalato-κ^2^ *O* ^1^:*O* ^2^-bis­[(dimethyl sulfoxide-κ*O*)tri­phenyl­tin(IV)]

**DOI:** 10.1107/S2056989017008519

**Published:** 2017-06-13

**Authors:** Serigne Fallou Pouye, Ibrahima Cisse, Libasse Diop, Alessandro Dolmella, Sylvain Bernès

**Affiliations:** a Laboratoire de Chimie Minérale et Analytique, Département de Chimie, Faculté des Sciences et Techniques, Université Cheikh Anta Diop, Dakar, Senegal; b Universita di Padova, Dipartimento di Scienze del Farmaco RMXS, Laboratorio di Radiofarmacia, Modellistica Molecolare e Diffrattometria a Raggi X, Via Francesco Marzolo 5, 35131, Padova, Italy; c Instituto de Física, Benemérita Universidad Autónoma de Puebla, Av. San Claudio y 18 Sur, 72570 Puebla, Pue., Mexico

**Keywords:** crystal structure, tin, oxalate, bridging ligand, disorder

## Abstract

In the previously reported [C_2_O_4_(SnPh_3_)_2_] complex [Diop *et al.* (2003 ▸). *Appl. Organomet. Chem.* **17**, 881–882.], the Sn^IV^ atoms are able to formally complete their coordination by addition of dimethyl sulfoxide (DMSO) mol­ecules provided by the reaction medium, affording the title complex, [Sn_2_(C_6_H_5_)_6_(C_2_O_4_)(C_2_H_6_OS)_2_]. The Sn^IV^ atoms are then penta­coordinated, with a common *trans* trigonal–bipyramidal arrangement. The asymmetric unit contains one half-mol­ecule, which is completed by inversion symmetry in space group type *C*2/*c*. The inversion centre is placed at the mid-point of the central bis-monodentate oxalate dianion, C_2_O_4_^2−^, which bridges the [(SnPh_3_)(DMSO)] moieties. The mol­ecule crystallizes as a disordered system, with two phenyl rings disordered by rotation about their Sn—C bonds, while the DMSO mol­ecule is split over two positions due to a tetra­hedral inversion at the S atom. All disordered parts were refined with occupancies fixed of 0.5.

## Abstract 2

The structure of the studied adduct consists of two dimethyl sulfoxide mol­ecules coordinating the [C_2_O_4_(SnPh_3_)_2_] core *via* their oxygen atoms. The Sn atoms display a *trans* trigonal–bipyramidal [SnC_3_O_2_] arrangement, and the oxalate dianion behaves as a bidentate bridging ligand.

## Chemical context   

One of the values of Sn^IV^ coordination chemistry is related to the ambiguous valency of this main element, for which a plethora of tetra- and penta­coordinated compounds have been described. This makes a difference with C and Si compounds, based almost exclusively on tetra­valent nodes, with very few cases of hypervalency. For Sn mononuclear compounds, a survey of the current Cambridge database (CSD V5.38 updated February 2017; Groom *et al.*, 2016 ▸) shows that coordination number four is more represented than coordination number five, with distributions of 63 and 37%, respectively, for the *ca* 4700 structures deposited to date. Stable compounds with a coordination number of four for the Sn^IV^ atom are thus attractive starting materials for the chemistry of Sn^IV^ complexes with a coordination number of five, including polynuclear species, which have no equivalent with the other elements of group 14. Tri­phenyl­tin chloride, SnPh_3_Cl, is one of these well used mol­ecules, with the additional advantage that the Cl atom may behave as a leaving group, while the SnPh_3_ fragment is a stable core structure.

The here reported dinuclear compound is a continuation of previous works carried out by the Dakar group about the synthesis of Sn_2_ complexes using the oxalate dianion as a bridging ligand. The simplest member of this family is [C_2_O_4_(SnPh_3_)_2_], where both Sn sites exhibit coordination number four (Diop *et al.*, 2003 ▸). However, it seems that whenever possible, the fifth coordination site in such complexes is occupied by a Lewis base, for example if the reaction is realized in a donor solvent such as H_2_O, DMF, thio­urea, *etc*. In this context, the structures of {C_2_O_4_[(SnMe_3_)(H_2_O)]_2_}, {C_2_O_4_[(SnPh_3_)(DMF)]_2_} and {C_2_O_4_[(SnPh_3_)(thio­urea)]_2_} have been described (Diop *et al.*, 1997 ▸; Gueye *et al.*, 2011 ▸; Sow *et al.*, 2012 ▸). In this dynamic, we now report a new complex synthesized using a mixture of dimethyl sulfoxide (DMSO) and methanol as solvent. The former component of this mixture is clearly a more stabilizing ligand for Sn atoms, resulting in the crystallization of the title compound, {C_2_O_4_[(SnPh_3_)(DMSO)]_2_}. Inter­estingly, the complex [(DMSO)SnPh_3_] is known (Kumar *et al.*, 2009 ▸), but was not detected in this reaction, indicating that the oxalate-bridged species is probably formed prior to solvent coordination.
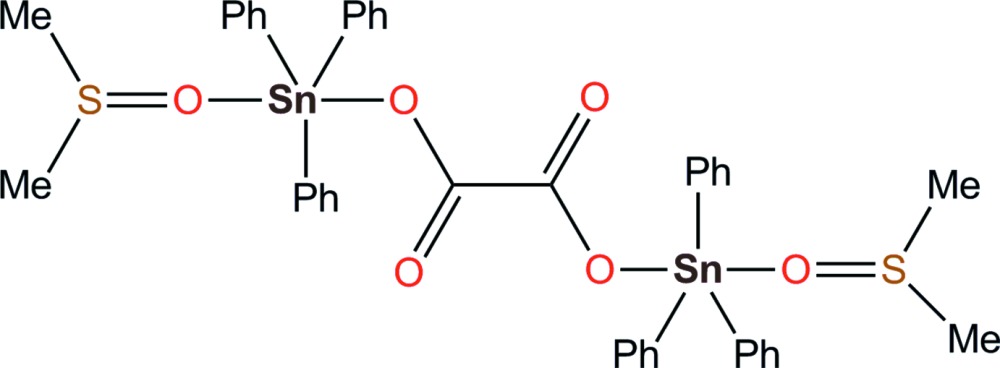


## Structural commentary   

**Figure 1:**
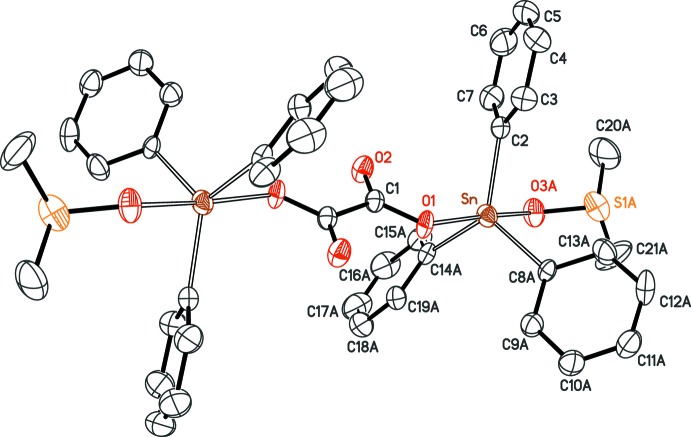
The mol­ecular structure of the title complex, with displacement ellipsoids at the 30% probability level. For phenyl rings C8–C13 and C14–C19 and for the DMSO mol­ecule, only disordered part *A* (occupancy 0.5) is represented, and all H atoms are omitted. Unlabelled atoms are generated by the symmetry operation (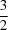 − *x*, 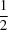 − *y*, 2 − *z*).

As expected, the oxalate dianion behaves as a bis-monodentate μ_2_-bridging ligand for two [SnPh_3_(DMSO)] moieties. The resulting neutral dinuclear complex is situated on a crystallographic inversion centre, placed at the midpoint of the C—C bond of the oxalate bridge (Fig. 1 ▸). Although that symmetry is consistent with the mol­ecular symmetry, the mol­ecule is strongly disordered: two of the three phenyl rings in the asymmetric unit are disordered over two positions by rotation about their Sn—C bonds, and the DMSO mol­ecule is also disordered over two positions, as a consequence of an inversion at the tetra­hedral S atom.

The Sn^IV^ atom is penta­coordinated in a common *trans* trigonal–bipyramidal manner, the phenyl groups being in equatorial positions, while the coordinating O atoms from the oxalate and DMSO ligands occupy the apical sites. The three Sn—C bonds are similar in length to those already reported for related complexes including the SnPh_3_ fragment (Sow *et al.*, 2012 ▸; Gueye *et al.*, 2011 ▸), while the Sn—O bond for the oxalate is rather short, 2.147 (2) Å, compared to that found in {C_2_O_4_[(SnMe_3_)(H_2_O)]_2_}, 2.209 Å, or in the anionic polymer [(CH_3_)_3_S]_*n*_[C_2_O_4_SnPh_3_]_*n*_ (2.220 Å; Ng *et al.*, 1994 ▸). This tight bonding character for the bridging oxalate may be related to its planar conformation, imposed by symmetry. The staggered arrangement for the six phenyl rings, also imposed by symmetry, avoids any steric hindrance in the complex. The apical DMSO mol­ecule has an Sn—O bond length of 2.354 (6)–2.403 (6) Å, reflecting a less pronounced coordination strength.

## Supra­molecular features   

**Figure 2:**
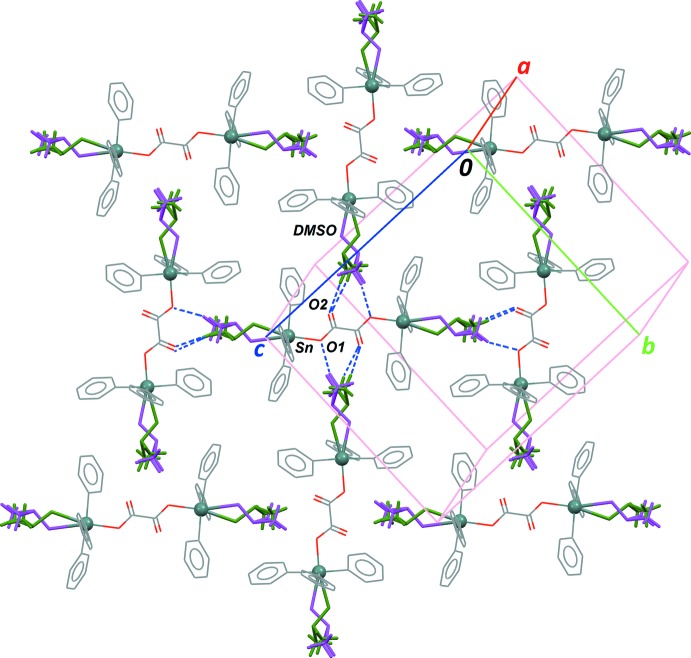
Part of the crystal structure viewed down reciprocal axis a*. For disordered phenyl rings, only one orientation is retained, while both disordered parts for the DMSO mol­ecules are represented, in green and magenta (parts *A* and *B*, respectively). Inter­molecular C—H⋯O contacts listed in Table 1 ▸ are represented for the central mol­ecule as blue dashed lines.

These discrete binuclear mol­ecules inter­act through van der Waals forces in the crystal, and no strong inter­actions are observed. The carbonyl groups of the oxalate dianion, C1—O1 and C1=O2, are the unique potential acceptor groups for hydrogen bonding, and indeed, weak inter­molecular C—H⋯O contacts are formed (Table 1 ▸): two mol­ecules related by a glide plane are oriented almost perpendicular, in such a way that methyl groups of the terminal DMSO ligands in one mol­ecule form C—H⋯O contacts with the oxalate bridge of the other mol­ecule (Fig. 2 ▸). These contacts are favoured by the disorder affecting the DMSO ligands, and allow to pack the complexes densely in the crystal, even in the absence of any π–π contacts between the phenyl rings.

## Database survey   

According to the Cambridge Structural Database (CSD V5.38 updated February 2017; Groom *et al.*, 2016 ▸), eleven structures containing a bis-monodentate bridging μ_2_-oxalate linked to two Sn atoms have been characterized by X-ray diffraction. In addition to those already mentioned in the previous sections, a *cis* [C_2_O_4_(Sn*R*_3_)_2_] complex with bulky *R* groups has been reported (Tan *et al.*, 2014 ▸), as well as stannate complexes (Sow *et al.*, 2011 ▸; Ng & Kumar Das, 1990 ▸, 1993 ▸; Kruger *et al.*, 1976 ▸). Among these structures, the *trans* coordination mode for the oxalate bridge dominates. The oxalate dianion is, however, known for having a very rich coordination behaviour, and the μ_2_-κ^2^-*O*,*O*′ coordination mode observed in the title compound is not the most common. Limiting the survey to Sn compounds, the chelating bis-bidentate bridging mode is more represented (*i.e*. polynuclear complexes including the μ_2_-oxalato-κ^4^ *O* ^1^,*O* ^2^:*O* ^1′^,*O* ^2′^ bridge). In that case, the conformation of the bridge is invariably planar, while the μ_2_-κ^2^-*O*,*O*′ bridge may be planar or twisted.

## Synthesis and crystallization   

[CH_3_NH_2_(CH_2_)_2_NH_2_CH_3_]C_2_O_4_ (**L**) was obtained as a powder, on mixing the di­amine CH_3_NH(CH_2_)_2_NHCH_3_ with C_2_O_4_H_2_·2H_2_O in a 1:1 ratio (*v*/*v*) in water, and allowing the water to evaporate at 333 K. When 0.10 g (0.26 mmol) of SnPh_3_Cl in 15 ml of a 1:1 ratio (*v*/*v*) DMSO/methanol mixture was reacted with 0.06 g (0.26 mmol) of **L**, a clear solution was obtained. Slow solvent evaporation over two weeks afforded a powder, which was collected. This powder dissolved in aceto­nitrile gave a slightly cloudy solution, which was quickly filtered off. The resulting clear solution, when allowed to evaporate slowly, afforded, six months after, colourless crystals of the title complex suitable for X-ray diffraction.

## Refinement   

Crystal data, data collection and structure refinement details are summarized in Table 2 ▸. The mol­ecular structure is strongly disordered. Three different data sets were collected for three different crystals, on different diffractometers; however, all gave the same disordered structure. In the asymmetric unit, two of the three phenyl rings are disordered over two positions: rings C8–C13 and C14–C19 were split over sites *A* and *B.* Attempts to refine site occupancies for the disordered parts resulted in free variables converging to values very close to 1/2 [maximum deviation for DMSO: 0.477 (5) and 0.523 (5)] with no clear improvement for the involved displacement parameters.

The four rings were restrained to be flat with standard deviation of 0.02 Å^3^, and the C atoms in a given ring were restrained to have the same anisotropic components, within a standard deviation of 0.04 Å^2^. Finally, *A* and *B* rings for each disordered phenyl group were restrained to have the same geometry (standard deviations: 0.02 Å for C C bond lengths and 0.04 Å for 1,3-distances). The DMSO mol­ecule is also disordered over two positions, labelled *A* and *B*, with occupancies fixed to 0.5. These parts were refined freely.

## Supplementary Material

Crystal structure: contains datablock(s) I, global. DOI: 10.1107/S2056989017008519/im2477sup1.cif

Structure factors: contains datablock(s) I. DOI: 10.1107/S2056989017008519/im2477Isup2.hkl

CCDC reference: 1554751

Additional supporting information:  crystallographic information; 3D view; checkCIF report

## Figures and Tables

**Table 1:** Hydrogen-bond geometry (Å, °)

*D*—H⋯*A*	*D*—H	H⋯*A*	*D*⋯*A*	*D*—H⋯*A*
C20*A*—H20*A*⋯O2^i^	0.96	2.51	3.43 (4)	162
C21*A*—H21*A*⋯O2^i^	0.96	2.60	3.49 (2)	154
C21*B*—H21*D*⋯O1^ii^	0.96	2.36	3.278 (19)	160

Symmetry codes: (i) 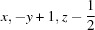; (ii) 
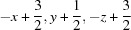
.

**Table 2:** Experimental details

Crystal data	
Chemical formula	[Sn_2_(C_6_H_5_)_6_(C_2_O_4_)(C_2_H_6_OS)_2_]
*M*_r_	944.25
Crystal system, space group	Monoclinic, *C*2/*c*
Temperature (K)	297
*a*, *b*, *c* (Å)	15.4638 (14), 16.2069 (10), 17.6205 (15)
β (°)	111.213 (10)
*V* (Å^3^)	4116.8 (6)
*Z*	4
Radiation type	Mo *K*α
μ (mm^−1^)	1.36
Crystal size (mm)	0.19 × 0.18 × 0.11

Data collection	
Diffractometer	Agilent Xcalibur Atlas Gemini
Absorption correction	Gaussian (*CrysAlis PRO*; Agilent, 2013 ▸)
*T*_min_, *T*_max_	0.955, 0.978
No. of measured, independent and observed [*I* > 2σ(*I*)] reflections	27591, 5762, 3476
*R*_int_	0.064
(sin θ/λ)_max_ (Å^−1^)	0.694

Refinement	
*R*[*F* ^2^ > 2σ(*F* ^2^)], *wR*(*F* ^2^), *S*	0.047, 0.090, 1.02
No. of reflections	5762
No. of parameters	383
No. of restraints	180
H-atom treatment	H-atom parameters constrained
Δρ_max_, Δρ_min_ (e Å^−3^)	0.50, −0.53

Computer programs: *CrysAlis PRO* (Agilent, 2013 ▸), *SHELXT2014* (Sheldrick, 2015*a* ▸), *SHELXL2016* (Sheldrick, 2015*b* ▸), *XP* in *SHELXTL* (Sheldrick, 2008 ▸) and *Mercury* (Macrae *et al.*, 2008 ▸).

## supplementary crystallographic information

### Crystal data

**Table:**

[Sn_2_(C_6_H_5_)_6_(C_2_O_4_)(C_2_H_6_OS)_2_]	*F*(000) = 1896
*M**_r_* = 944.25	*D*_x_ = 1.523 Mg m^−^^3^
Monoclinic, *C*2/*c*	Mo *K*α radiation, λ = 0.71073 Å
*a* = 15.4638 (14) Å	Cell parameters from 5828 reflections
*b* = 16.2069 (10) Å	θ = 3.5–25.5°
*c* = 17.6205 (15) Å	µ = 1.36 mm^−^^1^
β = 111.213 (10)°	*T* = 297 K
*V* = 4116.8 (6) Å^3^	Block, colourless
*Z* = 4	0.19 × 0.18 × 0.11 mm

### Data collection

**Table:**

Agilent Xcalibur Atlas Gemini diffractometer	5762 independent reflections
Radiation source: Enhance (Mo) X-ray Source	3476 reflections with *I* > 2σ(*I*)
Graphite monochromator	*R*_int_ = 0.064
Detector resolution: 10.5564 pixels mm^-1^	θ_max_ = 29.6°, θ_min_ = 3.0°
ω scans	*h* = −21→20
Absorption correction: gaussian (CrysAlis PRO; Agilent, 2013)	*k* = −20→22
*T*_min_ = 0.955, *T*_max_ = 0.978	*l* = −23→24
27591 measured reflections	

### Refinement

**Table:**

Refinement on *F*^2^	Primary atom site location: structure-invariant direct methods
Least-squares matrix: full	Secondary atom site location: difference Fourier map
*R*[*F*^2^ > 2σ(*F*^2^)] = 0.047	Hydrogen site location: inferred from neighbouring sites
*wR*(*F*^2^) = 0.090	H-atom parameters constrained
*S* = 1.02	*w* = 1/[σ^2^(*F*_o_^2^) + (0.0254*P*)^2^ + 2.4676*P*] where *P* = (*F*_o_^2^ + 2*F*_c_^2^)/3
5762 reflections	(Δ/σ)_max_ = 0.001
383 parameters	Δρ_max_ = 0.50 e Å^−^^3^
180 restraints	Δρ_min_ = −0.53 e Å^−^^3^
0 constraints	

### Fractional atomic coordinates and isotropic or equivalent isotropic displacement parameters (Å^2^)

**Table:**

	*x*	*y*	*z*	*U*_iso_*/*U*_eq_	Occ. (<1)
Sn	0.74863 (2)	0.38396 (2)	0.82897 (2)	0.05437 (10)	
O1	0.73200 (18)	0.28181 (13)	0.90012 (13)	0.0554 (6)	
O2	0.81455 (18)	0.34129 (15)	1.01798 (14)	0.0627 (7)	
C1	0.7646 (2)	0.2865 (2)	0.9783 (2)	0.0454 (8)	
C2	0.8950 (3)	0.3837 (2)	0.8639 (2)	0.0558 (9)	
C3	0.9412 (3)	0.3172 (3)	0.8478 (3)	0.0744 (12)	
H3A	0.908158	0.269872	0.824646	0.089*	
C4	1.0369 (4)	0.3197 (4)	0.8656 (3)	0.0940 (15)	
H4A	1.067176	0.274125	0.854909	0.113*	
C5	1.0860 (4)	0.3893 (4)	0.8988 (3)	0.1056 (19)	
H5A	1.149565	0.391281	0.910245	0.127*	
C6	1.0420 (4)	0.4552 (4)	0.9148 (3)	0.1067 (18)	
H6A	1.075612	0.502164	0.938234	0.128*	
C7	0.9470 (3)	0.4532 (3)	0.8967 (3)	0.0811 (13)	
H7A	0.917543	0.499630	0.906752	0.097*	
C8A	0.6690 (7)	0.3048 (6)	0.7188 (8)	0.041 (3)	0.5
C9A	0.5839 (5)	0.2732 (4)	0.7073 (4)	0.060 (2)	0.5
H9AA	0.558300	0.282628	0.746799	0.072*	0.5
C10A	0.5333 (6)	0.2272 (5)	0.6388 (5)	0.072 (2)	0.5
H10A	0.475126	0.206430	0.632941	0.087*	0.5
C11A	0.5698 (11)	0.2132 (8)	0.5811 (8)	0.073 (4)	0.5
H11A	0.536487	0.182846	0.534959	0.088*	0.5
C12A	0.6553 (12)	0.2434 (11)	0.5901 (11)	0.081 (5)	0.5
H12A	0.680493	0.232811	0.550534	0.097*	0.5
C13A	0.7046 (10)	0.2895 (10)	0.6578 (11)	0.069 (5)	0.5
H13A	0.762268	0.310741	0.662866	0.083*	0.5
C8B	0.6772 (8)	0.3329 (6)	0.7227 (8)	0.045 (3)	0.5
C9B	0.5824 (6)	0.3406 (6)	0.6914 (4)	0.074 (2)	0.5
H9BA	0.554540	0.375035	0.718110	0.089*	0.5
C10B	0.5261 (7)	0.2996 (7)	0.6223 (5)	0.100 (3)	0.5
H10B	0.462179	0.306921	0.603236	0.120*	0.5
C11B	0.5651 (13)	0.2487 (8)	0.5825 (9)	0.098 (5)	0.5
H11B	0.527747	0.220670	0.536246	0.118*	0.5
C12B	0.6595 (13)	0.2385 (12)	0.6105 (11)	0.089 (6)	0.5
H12B	0.686415	0.203745	0.583203	0.107*	0.5
C13B	0.7142 (11)	0.2802 (10)	0.6793 (11)	0.066 (5)	0.5
H13B	0.778203	0.273077	0.697646	0.079*	0.5
C14A	0.6762 (6)	0.4614 (6)	0.8894 (5)	0.051 (3)	0.5
C15A	0.6973 (8)	0.5429 (6)	0.9128 (5)	0.069 (3)	0.5
H15A	0.747612	0.568106	0.905281	0.083*	0.5
C16A	0.6444 (9)	0.5870 (10)	0.9473 (7)	0.097 (6)	0.5
H16A	0.658909	0.641741	0.962574	0.116*	0.5
C17A	0.5707 (8)	0.5503 (8)	0.9590 (6)	0.117 (4)	0.5
H17A	0.536018	0.580073	0.983054	0.141*	0.5
C18A	0.5470 (6)	0.4700 (7)	0.9358 (5)	0.099 (3)	0.5
H18A	0.495829	0.445820	0.942794	0.118*	0.5
C19A	0.6004 (5)	0.4259 (5)	0.9020 (4)	0.065 (2)	0.5
H19A	0.585513	0.371091	0.887310	0.078*	0.5
C14B	0.6686 (6)	0.4733 (6)	0.8548 (5)	0.048 (2)	0.5
C15B	0.6971 (7)	0.5049 (6)	0.9333 (6)	0.066 (3)	0.5
H15B	0.750368	0.484317	0.973173	0.079*	0.5
C16B	0.6469 (10)	0.5669 (8)	0.9528 (10)	0.098 (7)	0.5
H16B	0.666347	0.587680	1.005548	0.117*	0.5
C17B	0.5690 (8)	0.5971 (5)	0.8944 (8)	0.093 (3)	0.5
H17B	0.535329	0.638907	0.907265	0.111*	0.5
C18B	0.5398 (6)	0.5661 (6)	0.8164 (6)	0.095 (3)	0.5
H18B	0.486350	0.587047	0.777086	0.114*	0.5
C19B	0.5887 (5)	0.5041 (5)	0.7957 (5)	0.066 (2)	0.5
H19B	0.568470	0.483432	0.742912	0.079*	0.5
S1A	0.7716 (2)	0.52338 (16)	0.68682 (18)	0.0811 (8)	0.5
O3A	0.7554 (4)	0.5126 (4)	0.7693 (4)	0.0732 (16)	0.5
C20A	0.865 (2)	0.575 (3)	0.708 (3)	0.137 (14)	0.5
H20A	0.864484	0.604834	0.660865	0.205*	0.5
H20B	0.917345	0.538392	0.725717	0.205*	0.5
H20C	0.870035	0.613586	0.751115	0.205*	0.5
C21A	0.6862 (16)	0.5830 (16)	0.6302 (14)	0.184 (13)	0.5
H21A	0.701270	0.605139	0.585978	0.276*	0.5
H21B	0.677325	0.627345	0.662722	0.276*	0.5
H21C	0.630197	0.551118	0.608857	0.276*	0.5
S1B	0.76364 (17)	0.56427 (15)	0.72611 (17)	0.0723 (6)	0.5
O3B	0.7508 (4)	0.4700 (3)	0.7186 (4)	0.0728 (17)	0.5
C20B	0.8649 (16)	0.597 (3)	0.710 (3)	0.096 (7)	0.5
H20D	0.861031	0.655418	0.699420	0.144*	0.5
H20E	0.870286	0.568154	0.664730	0.144*	0.5
H20F	0.918280	0.585965	0.758107	0.144*	0.5
C21B	0.6869 (12)	0.6082 (12)	0.6425 (14)	0.101 (6)	0.5
H21D	0.704256	0.664491	0.639061	0.152*	0.5
H21E	0.626177	0.606439	0.645634	0.152*	0.5
H21F	0.686267	0.578628	0.595114	0.152*	0.5

### Atomic displacement parameters (Å^2^)

**Table:**

	*U*^11^	*U*^22^	*U*^33^	*U*^12^	*U*^13^	*U*^23^
Sn	0.05831 (17)	0.05370 (17)	0.05635 (17)	0.00491 (13)	0.02705 (13)	0.01576 (12)
O1	0.0847 (18)	0.0497 (14)	0.0311 (13)	−0.0065 (12)	0.0201 (12)	0.0076 (10)
O2	0.0827 (19)	0.0523 (15)	0.0485 (15)	−0.0168 (14)	0.0183 (14)	0.0011 (12)
C1	0.055 (2)	0.0419 (19)	0.041 (2)	0.0038 (17)	0.0200 (17)	0.0037 (15)
C2	0.055 (2)	0.072 (3)	0.043 (2)	−0.002 (2)	0.0201 (17)	0.0074 (18)
C3	0.064 (3)	0.084 (3)	0.075 (3)	0.002 (2)	0.024 (2)	0.000 (2)
C4	0.074 (4)	0.125 (5)	0.091 (4)	0.017 (3)	0.040 (3)	−0.001 (3)
C5	0.063 (3)	0.174 (6)	0.083 (4)	−0.025 (4)	0.030 (3)	−0.021 (4)
C6	0.088 (4)	0.145 (5)	0.079 (4)	−0.042 (4)	0.021 (3)	−0.023 (3)
C7	0.079 (3)	0.095 (4)	0.067 (3)	−0.009 (3)	0.024 (2)	−0.012 (2)
C8A	0.054 (6)	0.033 (6)	0.032 (4)	−0.004 (4)	0.011 (4)	0.007 (4)
C9A	0.063 (5)	0.063 (5)	0.053 (5)	−0.008 (4)	0.021 (4)	−0.004 (4)
C10A	0.071 (6)	0.078 (6)	0.064 (6)	−0.026 (5)	0.021 (5)	−0.005 (5)
C11A	0.085 (8)	0.074 (8)	0.052 (6)	−0.020 (6)	0.014 (6)	−0.001 (5)
C12A	0.126 (13)	0.083 (10)	0.046 (7)	−0.007 (9)	0.045 (8)	−0.006 (6)
C13A	0.072 (9)	0.075 (9)	0.073 (11)	−0.019 (7)	0.041 (8)	0.004 (7)
C8B	0.053 (6)	0.047 (8)	0.037 (5)	−0.005 (5)	0.020 (4)	0.007 (5)
C9B	0.061 (6)	0.113 (7)	0.047 (5)	−0.006 (6)	0.017 (4)	−0.014 (5)
C10B	0.062 (6)	0.169 (11)	0.060 (6)	−0.019 (7)	0.010 (5)	−0.025 (7)
C11B	0.108 (11)	0.125 (14)	0.052 (7)	−0.027 (10)	0.016 (7)	−0.023 (8)
C12B	0.117 (13)	0.085 (11)	0.068 (11)	0.013 (9)	0.036 (8)	−0.011 (8)
C13B	0.070 (8)	0.065 (8)	0.057 (8)	0.021 (6)	0.018 (6)	−0.005 (6)
C14A	0.059 (5)	0.056 (6)	0.032 (5)	0.002 (4)	0.010 (5)	0.002 (5)
C15A	0.074 (6)	0.061 (7)	0.063 (7)	0.021 (6)	0.013 (5)	−0.008 (5)
C16A	0.100 (14)	0.082 (9)	0.100 (12)	0.022 (8)	0.026 (11)	−0.038 (8)
C17A	0.095 (9)	0.155 (12)	0.091 (8)	0.038 (9)	0.020 (7)	−0.045 (8)
C18A	0.074 (6)	0.152 (10)	0.076 (6)	0.008 (7)	0.035 (5)	−0.012 (6)
C19A	0.065 (5)	0.084 (6)	0.051 (5)	0.003 (4)	0.027 (4)	−0.005 (4)
C14B	0.053 (5)	0.049 (5)	0.045 (6)	−0.001 (4)	0.021 (5)	0.003 (4)
C15B	0.067 (6)	0.060 (7)	0.065 (7)	0.023 (6)	0.018 (5)	0.013 (6)
C16B	0.101 (14)	0.073 (9)	0.106 (12)	0.022 (8)	0.021 (10)	−0.020 (7)
C17B	0.091 (8)	0.071 (7)	0.130 (10)	0.022 (6)	0.057 (8)	−0.004 (6)
C18B	0.064 (6)	0.100 (8)	0.111 (8)	0.028 (5)	0.019 (6)	0.021 (6)
C19B	0.058 (5)	0.078 (6)	0.059 (5)	0.006 (4)	0.019 (4)	0.004 (4)
S1A	0.110 (2)	0.0488 (14)	0.107 (2)	−0.0064 (13)	0.0655 (18)	−0.0066 (14)
O3A	0.103 (5)	0.057 (4)	0.069 (4)	−0.005 (3)	0.043 (4)	0.021 (3)
C20A	0.125 (19)	0.15 (3)	0.152 (19)	−0.008 (14)	0.076 (15)	0.07 (2)
C21A	0.128 (16)	0.27 (3)	0.093 (12)	−0.073 (16)	−0.037 (10)	0.093 (16)
S1B	0.0840 (17)	0.0567 (14)	0.0848 (18)	−0.0119 (12)	0.0407 (14)	−0.0040 (13)
O3B	0.108 (5)	0.039 (3)	0.084 (5)	−0.010 (3)	0.050 (4)	0.010 (3)
C20B	0.045 (9)	0.110 (13)	0.125 (14)	−0.016 (7)	0.021 (8)	0.059 (11)
C21B	0.080 (10)	0.080 (7)	0.171 (17)	0.044 (7)	0.079 (11)	0.071 (9)

### Geometric parameters (Å, º)

**Table:**

Sn—C8B	1.979 (13)	C12B—H12B	0.9300
Sn—C14B	2.060 (10)	C13B—H13B	0.9300
Sn—C2	2.120 (4)	C14A—C15A	1.387 (11)
Sn—O1	2.147 (2)	C14A—C19A	1.394 (10)
Sn—C14A	2.196 (10)	C15A—C16A	1.383 (12)
Sn—C8A	2.281 (12)	C15A—H15A	0.9300
Sn—O3A	2.354 (6)	C16A—C17A	1.366 (15)
Sn—O3B	2.403 (6)	C16A—H16A	0.9300
O1—C1	1.286 (4)	C17A—C18A	1.373 (12)
O2—C1	1.218 (4)	C17A—H17A	0.9300
C1—C1^i^	1.562 (6)	C18A—C19A	1.381 (10)
C2—C3	1.379 (5)	C18A—H18A	0.9300
C2—C7	1.382 (6)	C19A—H19A	0.9300
C3—C4	1.398 (6)	C14B—C19B	1.388 (10)
C3—H3A	0.9300	C14B—C15B	1.389 (11)
C4—C5	1.367 (7)	C15B—C16B	1.387 (12)
C4—H4A	0.9300	C15B—H15B	0.9300
C5—C6	1.350 (7)	C16B—C17B	1.361 (14)
C5—H5A	0.9300	C16B—H16B	0.9300
C6—C7	1.386 (6)	C17B—C18B	1.378 (12)
C6—H6A	0.9300	C17B—H17B	0.9300
C7—H7A	0.9300	C18B—C19B	1.384 (10)
C8A—C9A	1.357 (11)	C18B—H18B	0.9300
C8A—C13A	1.395 (12)	C19B—H19B	0.9300
C9A—C10A	1.394 (9)	S1A—O3A	1.572 (6)
C9A—H9AA	0.9300	S1A—C20A	1.60 (3)
C10A—C11A	1.348 (12)	S1A—C21A	1.65 (2)
C10A—H10A	0.9300	C20A—H20A	0.9600
C11A—C12A	1.365 (13)	C20A—H20B	0.9600
C11A—H11A	0.9300	C20A—H20C	0.9600
C12A—C13A	1.380 (12)	C21A—H21A	0.9600
C12A—H12A	0.9300	C21A—H21B	0.9600
C13A—H13A	0.9300	C21A—H21C	0.9600
C8B—C9B	1.372 (11)	S1B—O3B	1.540 (6)
C8B—C13B	1.399 (11)	S1B—C21B	1.679 (19)
C9B—C10B	1.384 (10)	S1B—C20B	1.77 (3)
C9B—H9BA	0.9300	C20B—H20D	0.9600
C10B—C11B	1.357 (13)	C20B—H20E	0.9600
C10B—H10B	0.9300	C20B—H20F	0.9600
C11B—C12B	1.371 (13)	C21B—H21D	0.9600
C11B—H11B	0.9300	C21B—H21E	0.9600
C12B—C13B	1.379 (12)	C21B—H21F	0.9600
			
C8B—Sn—C2	116.5 (4)	C8B—C13B—H13B	118.7
C14B—Sn—C2	126.9 (3)	C15A—C14A—C19A	117.8 (9)
C8B—Sn—O1	94.9 (3)	C15A—C14A—Sn	125.6 (7)
C14B—Sn—O1	101.8 (2)	C19A—C14A—Sn	116.6 (7)
C2—Sn—O1	99.80 (12)	C16A—C15A—C14A	120.7 (11)
C2—Sn—C14A	122.6 (3)	C16A—C15A—H15A	119.6
O1—Sn—C14A	88.1 (2)	C14A—C15A—H15A	119.6
C2—Sn—C8A	115.9 (3)	C17A—C16A—C15A	119.9 (13)
O1—Sn—C8A	85.5 (3)	C17A—C16A—H16A	120.0
C2—Sn—O3A	85.17 (18)	C15A—C16A—H16A	120.0
O1—Sn—O3A	167.81 (17)	C16A—C17A—C18A	121.1 (11)
C2—Sn—O3B	84.84 (18)	C16A—C17A—H17A	119.5
O1—Sn—O3B	163.62 (16)	C18A—C17A—H17A	119.5
C1—O1—Sn	119.8 (2)	C17A—C18A—C19A	118.8 (9)
O2—C1—O1	125.4 (3)	C17A—C18A—H18A	120.6
O2—C1—C1^i^	120.4 (4)	C19A—C18A—H18A	120.6
O1—C1—C1^i^	114.2 (4)	C18A—C19A—C14A	121.6 (9)
C3—C2—C7	117.2 (4)	C18A—C19A—H19A	119.2
C3—C2—Sn	121.3 (3)	C14A—C19A—H19A	119.2
C7—C2—Sn	121.2 (3)	C19B—C14B—C15B	119.3 (9)
C2—C3—C4	121.1 (4)	C19B—C14B—Sn	122.0 (6)
C2—C3—H3A	119.5	C15B—C14B—Sn	118.7 (7)
C4—C3—H3A	119.5	C16B—C15B—C14B	120.6 (10)
C5—C4—C3	119.9 (5)	C16B—C15B—H15B	119.7
C5—C4—H4A	120.0	C14B—C15B—H15B	119.7
C3—C4—H4A	120.0	C17B—C16B—C15B	119.7 (12)
C6—C5—C4	119.9 (5)	C17B—C16B—H16B	120.2
C6—C5—H5A	120.1	C15B—C16B—H16B	120.2
C4—C5—H5A	120.1	C16B—C17B—C18B	120.2 (10)
C5—C6—C7	120.3 (5)	C16B—C17B—H17B	119.9
C5—C6—H6A	119.8	C18B—C17B—H17B	119.9
C7—C6—H6A	119.8	C17B—C18B—C19B	121.1 (8)
C2—C7—C6	121.6 (5)	C17B—C18B—H18B	119.5
C2—C7—H7A	119.2	C19B—C18B—H18B	119.5
C6—C7—H7A	119.2	C18B—C19B—C14B	119.1 (8)
C9A—C8A—C13A	116.7 (10)	C18B—C19B—H19B	120.5
C9A—C8A—Sn	122.4 (9)	C14B—C19B—H19B	120.5
C13A—C8A—Sn	120.9 (9)	O3A—S1A—C20A	105.9 (18)
C8A—C9A—C10A	122.8 (9)	O3A—S1A—C21A	105.3 (9)
C8A—C9A—H9AA	118.6	C20A—S1A—C21A	107.1 (17)
C10A—C9A—H9AA	118.6	S1A—O3A—Sn	124.0 (4)
C11A—C10A—C9A	119.0 (9)	S1A—C20A—H20A	109.5
C11A—C10A—H10A	120.5	S1A—C20A—H20B	109.5
C9A—C10A—H10A	120.5	H20A—C20A—H20B	109.5
C10A—C11A—C12A	120.3 (12)	S1A—C20A—H20C	109.5
C10A—C11A—H11A	119.9	H20A—C20A—H20C	109.5
C12A—C11A—H11A	119.9	H20B—C20A—H20C	109.5
C11A—C12A—C13A	120.3 (13)	S1A—C21A—H21A	109.5
C11A—C12A—H12A	119.9	S1A—C21A—H21B	109.5
C13A—C12A—H12A	119.9	H21A—C21A—H21B	109.5
C12A—C13A—C8A	120.9 (12)	S1A—C21A—H21C	109.5
C12A—C13A—H13A	119.6	H21A—C21A—H21C	109.5
C8A—C13A—H13A	119.6	H21B—C21A—H21C	109.5
C9B—C8B—C13B	115.2 (11)	O3B—S1B—C21B	108.4 (8)
C9B—C8B—Sn	119.4 (9)	O3B—S1B—C20B	112.3 (15)
C13B—C8B—Sn	125.0 (10)	C21B—S1B—C20B	96.8 (13)
C8B—C9B—C10B	123.3 (9)	S1B—O3B—Sn	123.0 (4)
C8B—C9B—H9BA	118.3	S1B—C20B—H20D	109.5
C10B—C9B—H9BA	118.3	S1B—C20B—H20E	109.5
C11B—C10B—C9B	119.4 (10)	H20D—C20B—H20E	109.5
C11B—C10B—H10B	120.3	S1B—C20B—H20F	109.5
C9B—C10B—H10B	120.3	H20D—C20B—H20F	109.5
C10B—C11B—C12B	120.2 (12)	H20E—C20B—H20F	109.5
C10B—C11B—H11B	119.9	S1B—C21B—H21D	109.5
C12B—C11B—H11B	119.9	S1B—C21B—H21E	109.5
C11B—C12B—C13B	119.4 (13)	H21D—C21B—H21E	109.5
C11B—C12B—H12B	120.3	S1B—C21B—H21F	109.5
C13B—C12B—H12B	120.3	H21D—C21B—H21F	109.5
C12B—C13B—C8B	122.5 (12)	H21E—C21B—H21F	109.5
C12B—C13B—H13B	118.7		
			
Sn—O1—C1—O2	−8.7 (5)	C11B—C12B—C13B—C8B	0.3 (16)
Sn—O1—C1—C1^i^	171.7 (3)	C9B—C8B—C13B—C12B	−0.4 (12)
C7—C2—C3—C4	1.3 (6)	Sn—C8B—C13B—C12B	172.0 (9)
Sn—C2—C3—C4	175.3 (3)	C19A—C14A—C15A—C16A	−0.2 (4)
C2—C3—C4—C5	−0.7 (7)	Sn—C14A—C15A—C16A	177.1 (6)
C3—C4—C5—C6	0.5 (8)	C14A—C15A—C16A—C17A	0.3 (5)
C4—C5—C6—C7	−1.1 (9)	C15A—C16A—C17A—C18A	−1.0 (11)
C3—C2—C7—C6	−1.9 (6)	C16A—C17A—C18A—C19A	1.6 (13)
Sn—C2—C7—C6	−175.8 (3)	C17A—C18A—C19A—C14A	−1.4 (11)
C5—C6—C7—C2	1.8 (8)	C15A—C14A—C19A—C18A	0.7 (9)
C13A—C8A—C9A—C10A	0.3 (5)	Sn—C14A—C19A—C18A	−176.8 (6)
Sn—C8A—C9A—C10A	177.7 (6)	C19B—C14B—C15B—C16B	−0.2 (5)
C8A—C9A—C10A—C11A	0.0 (6)	Sn—C14B—C15B—C16B	178.0 (6)
C9A—C10A—C11A—C12A	0.4 (12)	C14B—C15B—C16B—C17B	−0.1 (5)
C10A—C11A—C12A—C13A	−1.0 (16)	C15B—C16B—C17B—C18B	0.3 (10)
C11A—C12A—C13A—C8A	1.3 (16)	C16B—C17B—C18B—C19B	−0.2 (12)
C9A—C8A—C13A—C12A	−0.9 (12)	C17B—C18B—C19B—C14B	−0.1 (11)
Sn—C8A—C13A—C12A	−178.4 (9)	C15B—C14B—C19B—C18B	0.4 (9)
C13B—C8B—C9B—C10B	0.2 (5)	Sn—C14B—C19B—C18B	−177.8 (6)
Sn—C8B—C9B—C10B	−172.7 (7)	C20A—S1A—O3A—Sn	−119.3 (17)
C8B—C9B—C10B—C11B	0.3 (6)	C21A—S1A—O3A—Sn	127.4 (10)
C9B—C10B—C11B—C12B	−0.5 (12)	C21B—S1B—O3B—Sn	−135.9 (7)
C10B—C11B—C12B—C13B	0.2 (16)	C20B—S1B—O3B—Sn	118.3 (13)

Symmetry code: (i) −*x*+3/2, −*y*+1/2, −*z*+2.

### Hydrogen-bond geometry (Å, º)

**Table:**

*D*—H···*A*	*D*—H	H···*A*	*D*···*A*	*D*—H···*A*
C20*A*—H20*A*···O2^ii^	0.96	2.51	3.43 (4)	162
C21*A*—H21*A*···O2^ii^	0.96	2.60	3.49 (2)	154
C21*B*—H21*D*···O1^iii^	0.96	2.36	3.278 (19)	160

Symmetry codes: (ii) *x*, −*y*+1, *z*−1/2; (iii) −*x*+3/2, *y*+1/2, −*z*+3/2.
